# Case Report: Low-Dose Decitabine Plus Anti-PD-1 Inhibitor Camrelizumab for Previously Treated Advanced Metastatic Non-Small Cell Lung Cancer

**DOI:** 10.3389/fonc.2020.558572

**Published:** 2020-10-22

**Authors:** Xin Yan, Yongtian Zhao, Yang Liu, Qingming Yang, Liang Dong, Zhiqiang Wu, Jing Nie, Deyun Chen, Miaomiao Bai, Dongdong Ti, Kaichao Feng, Weidong Han

**Affiliations:** ^1^School of Medicine, Nankai University, Tianjin, China; ^2^Department of Bio-Therapeutic, The First Medical Centre, Chinese PLA General Hospital, Beijing, China; ^3^YuceBio, Shenzhen, China

**Keywords:** programmed cell death protein 1, immunotherapy, decitabine, treatment, epigenetics

## Abstract

**Background:** Although the programmed death 1 (PD-1)/programmed death-ligand 1 (PD-L1) inhibitors have markedly changed the strategies of cancer treatment, most patients with advanced non-small cell lung cancer (NSCLC) do not respond to PD-1/PD-L1 monotherapy. Epigenetic drugs have been hypothesized to possess the potential to sensitize PD-1/PD-L1 inhibitors.

**Case Presentation:** Three patients with advanced metastatic NSCLC failed to respond to first-line systemic therapy and had a low tumor mutation burden, low tumor neoantigen burden, low microsatellite instability, and HLA loss of heterozygosity according to their target lesion biopsies, all of which were considered unfavorable factors for PD-1/PD-L1 blockage. However, all three patients responded to low-dose decitabine, an epigenetic drug, in combination with camrelizumab (anti-PD-1 antibody), with only controllable adverse events, indicating that low-dose decitabine can sensitize PD-1/PD-L1 inhibitors.

**Summary:** We report a novel therapy with low-dose decitabine plus camrelizumab for advanced NSCLC on the basis of successful treatment of three patients, emphasizing the potential of epigenetic drugs to regulate PD-1/PD-L1 inhibitors in advanced NSCLC.

## Introduction

Programmed cell death protein 1(PD-1)/programmed cell death-ligand 1(PD-L1) inhibitors have changed the treatment strategy for lung cancer (1, 2). However, only some patients with advanced metastatic non-small cell lung cancer (NSCLC) can benefit from PD-1/PD-L1 blockade monotherapy. The combination of PD-1/PD-L1 inhibitors and other therapies has attracted increasing attention (3). Decitabine, a type of hypomethylating agent (HMA), is approved by the United States Food and Drug Administration for the treatment of myelodysplastic syndrome and acute myeloid leukemia (4). It has been reported that low-dose decitabine could increase CD8^+^, CD4^+^, and IFNγ^+^ T cell infiltration and broaden the peripheral T cell receptor repertoire through DNA demethylation (5–7). Therefore, it was hypothesized that low-dose decitabine as an epigenetic drug could improve the efficacy of PD-1/PD-L1 antibodies. Currently, epigenetic drugs in combination with PD-1/PD-L1 antibodies have been studied for the treatment of lymphoma, acute myeloid leukemia, and myelodysplastic syndrome (4, 8–10), but there are few studies on their efficacy against solid tumors.

In this study, we present three cases of previously treated advanced metastatic NSCLC that benefitted from low-dose decitabine plus PD-1 inhibitor camrelizumab therapy.

## Case Description

### Case 1

**Figure 1:**
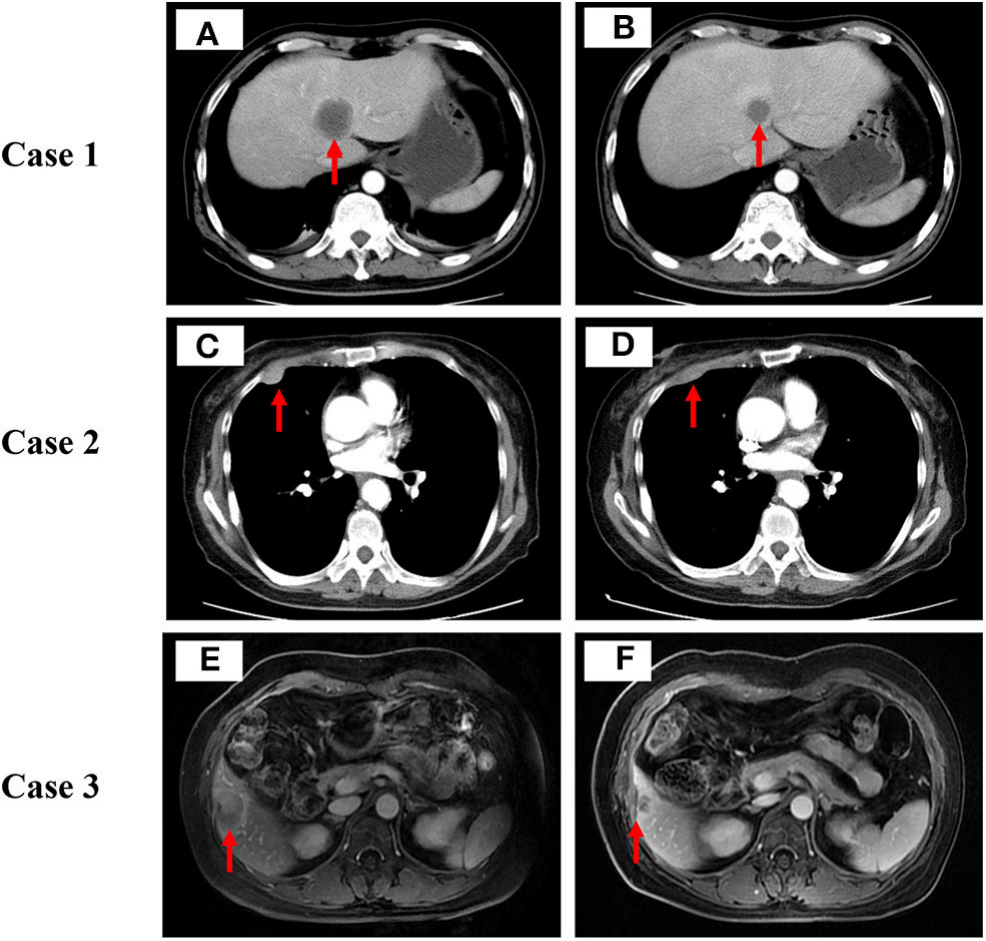
Computed tomography scan or magnetic resonance imaging scan before **(A,C,E)** and after **(B,D,F)** low-dose decitabine combined with camrelizumab therapy in these 3 cases. Tumor lesions are indicated by red arrows.

A 47-year-old man with no history of smoking was hospitalized due to irritating cough. The computed tomography (CT) scan showed a mass in the right lung with multiple metastases in mediastinal lymph nodes and right bronchopulmonary hilar lymph nodes. Pathology of right lung puncture was undifferentiated adenocarcinoma. Molecular testing of small biopsy specimens revealed no positive mutation or rearrangement of epidermal growth factor receptor (EGFR) and Anaplastic Lymphoma kinase (ALK). He was diagnosed with stage IV lung adenocarcinoma in November, 2016. Then he was treated with 6 cycles of first-line bevacizumab plus pemetrexed and cisplatin and proton radiation therapy. In August 2017, his CT scan showed multiple new metastatic lesions in bilateral supraclavicular lymph nodes, left axillary lymph nodes, retroperitoneal lymph nodes, bilateral adrenal glands, bones, and the liver, which had the largest target lesion (Figure 1A). Biopsy of the right supraclavicular metastatic lymph nodes revealed infiltration of poorly differentiated adenocarcinoma and negative molecular results. Exon sequencing results of the tumor tissue showed that the tumor mutation load (TMB) and tumor neoantigen burden (TNB) were 7.83 Muts/Mb and 3.72 Neos/Mb, respectively. Furthermore, the results revealed low microsatellite instability (MSI) and positivity for HLA loss of heterozygosity (HLA LOH). PD-L1 expression of tumor tissue measured based on tumor proportion score was positive.

In September 2017, the patient was prescribed low-dose decitabine (10 mg/day, days 1–5) combined with camrelizumab (200 mg, day 6) treatment every 3 weeks for 6 cycles. After 2 cycles of combination treatment, a CT scan showed that the number of mediastinal lymph nodes, right bronchopulmonary hilar lymph nodes, bilateral supraclavicular lymph nodes, left axillary lymph nodes, and retroperitoneal lymph nodes reduced significantly. The metastatic lesions in the bilateral adrenal glands, bones, and liver shrunk dramatically (Figure 1B). Irritating cough of the patient was alleviated significantly.

After 6 cycles of treatment, a new hypoechoic mass was found in the patient's back, which was confirmed from NSCLC metastasis by pathology. The patient discontinued low-dose decitabine combined with camrelizumab treatment due to disease progression. Tumor mutation load decreased from 7.83 to 0.1 Muts/Mb, TNB decreased from 3.72 to 0.03 Neos/Mb, and low MSI status and HLA-C LOH events remained unchanged during treatment. The patient complained of occasional mild nausea. The grade 3 anemia (serum hemoglobin: 70 g/L) and grade 2 nausea according to the National Cancer Institute Common Terminology Criteria for Adverse Events (CTCAE, version 4.0), was detected and could be rectified by symptomatic treatment. After the disease progressed, the patient was transitioned to hospice care and died of lung infection in April 2018.

### Case 2

A 74-year-old woman without a prior history of smoking was hospitalized for pain in the right lower chest wall. CT scan detected multiple nodules in the right lung accompany with mediastinal lymph nodes, liver, and pleural nodules metastases. Pathology of right lung mass suggests lung adenocarcinoma. Molecular testing of tumor tissues revealed *EGFR* 858R and *EGFR* T790M mutations. Results for other common carcinogenic gene mutations and rearrangements were negative. In April 2016, the patient was diagnosed with stage IV lung adenocarcinoma. She was prescribed gefitinib for 7 months, then she switched to osimertinib, which obtained stable disease in 1 month, and disease progress in 3 months. She was given pemetrexed for another 2 cycles, but discontinued the treatment due to excessive gastrointestinal reaction intolerance. Besides, she was also subjected to microwave ablation of the liver. In September 2017, a CT scan revealed enlarged pulmonary and pleural nodules (Figure 1C). Exon sequencing results of the metastatic pleural nodules revealed low TMB (2.49 Muts/Mb), low TNB (1.55 Neos/Mb), low MSI, and positivity for HLA LOH. In the same month, the patient received low-dose decitabine (10 mg/day, days 1–5) plus camrelizumab (200 mg, day 6) every 3 weeks for 7 cycles. The mediastinal lymph nodes, liver, and pleural nodule metastases dramatically shrunk after 2 cycles of combination therapy (Figure 1D). Besides, her pain in the right lower chest wall also improved obviously after 2 cycles of treatment. After 7 cycles of treatment, the lung CT scan showed enlargement of the right lung and pleural lesions. She stopped low-dose decitabine combined with camrelizumab treatment due to disease progression. During combined therapy, TMB increased from 2.49 to 2.98 Muts/Mb, TNB decreased from 1.55 to 1.32 Neos/Mb, and the MSI status and HLA LOH events remained unchanged. The patient admitted to having moderate fatigue and vomit. The clinician assessed that he had experienced grade 2 fatigue and grade 2 vomit in terms of the CTCAE (version 4.0), and the adverse events were tolerable. Finally, the patient died of multiple organ failure in May 2019.

### Case 3

A 46-year-old female patient with no smoking history sought treatment due to tussiculation. CT scan found a mass in the upper left hilum with bilateral mediastinal lymph nodes, liver, and bone metastases. Tumor biopsy (bronchoscopy) pathology revealed invasive lung adenocarcinoma in the upper left lobe. No positive mutation or rearrangement of EGFR or ALK was found on molecular testing of small biopsy specimens. PD-L1 expression of tumor tissue measured based on tumor proportion score was positive. The patient was diagnosed IV lung adenocarcinoma in October 2015. She was administered 18 cycles of pemetrexed plus cisplatin and bevacizumab and was subjected to thrombectomy for hepatic artery chemotherapy. In April 2017, a CT scan revealed that metastatic lesions in the liver increased significantly (Figure 1E) and thus the patient was administered low-dose decitabine (10 mg/day, days 1–5) plus camrelizumab (200 mg, day 6) every 3 weeks for 8 cycles. After 2 cycles of treatment, abdominal magnetic resonance imaging revealed a reduction in the lesions of the liver lobe (Figure 1F). What's more, tussiculation of the patient also improved significantly. After 8 cycles of therapy, the patient showed enlarged liver lesions. She stopped combination therapy as a result of disease progression. Simultaneously, TMB decreased from 1.91 to 1.16 Muts/Mb, TNB decreased from 0.75 to 0.45 Neos/Mb, and MSI status and HLA LOH events remained unchanged. The patient complained of occasional mild decreased appetite. Physician assessed him to have controllable grade 2 hyperthyroidism and grade 2 fatigue based on the CTCAE (version 4.0) during combination therapy. After disease progression, she switched to crizotinib orally because ALK gene mutation was detected.

**Figure 2:**
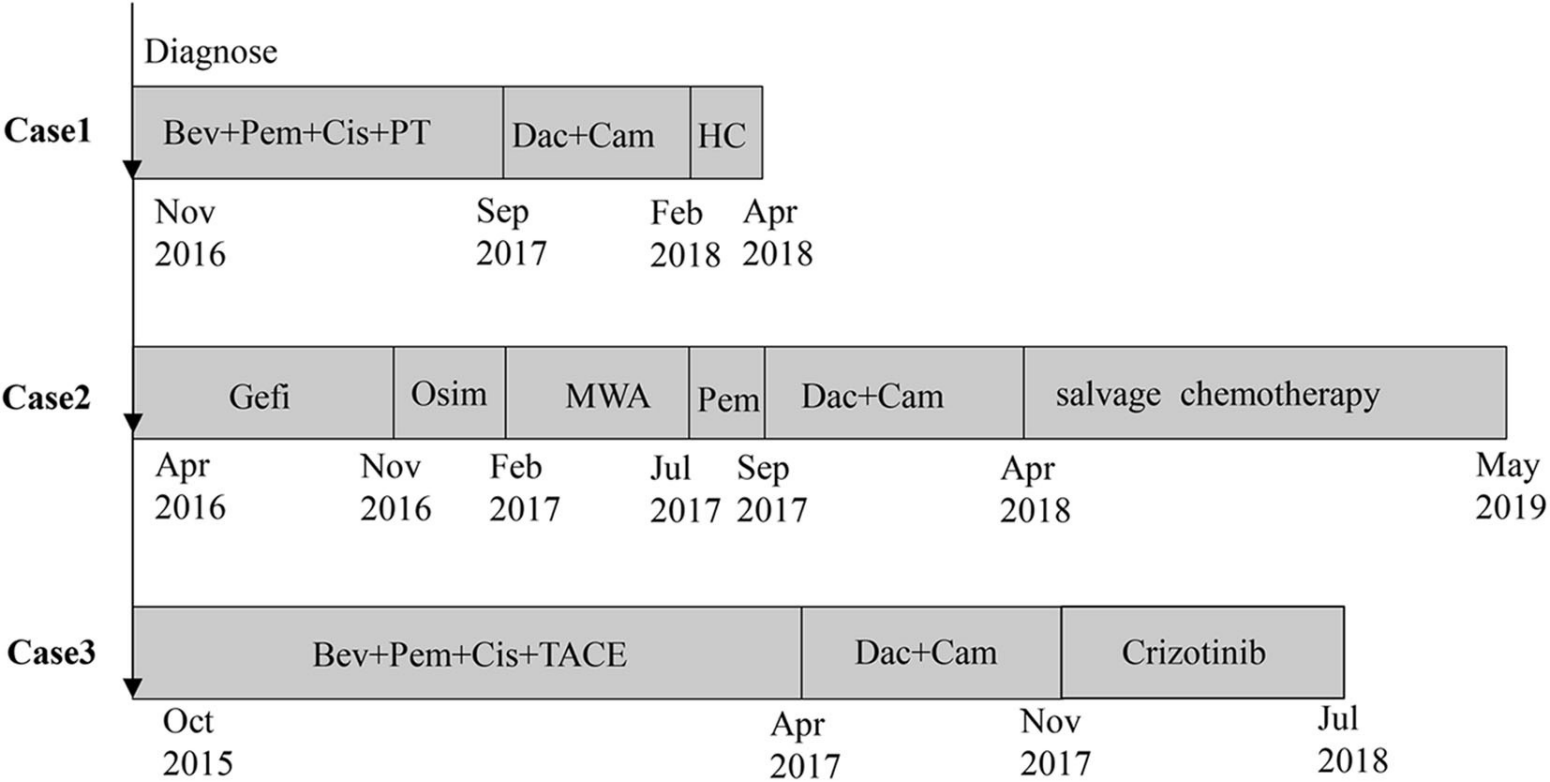
The treatment timeline of the three patients.

All the three patients' characteristics, whole exon sequencing results (MagBind® Blood & Tissue DNA HDQ 96 Kit, Agilent Technologies) of tumor biopsies before and after combination therapy are shown in Table 1 A figure of the treatment timeline of the three patients is revealed in Figure 2. The expression of PD-L1 on tumor cells was evaluated by tumor proportion score using the Dako 22C3 pharmDx assay (Dako North America, Carpinteria, California, USA) on archives or fresh pre-treated biopsy samples. PD-L1 expression of tumor tissue measured based on tumor proportion score. Tumor PD-L1 positive expression means that at least 1% of tumor cells are enveloped with any intensity in at least 100 sections where tumor cells can be evaluated. All three patients signed an informed consent and were included in the clinical trial of decitabine combined with anti-PD-1 antibody (NCT01799083).

**Table 1:** Patient characteristics and whole exon sequencing results of tumor biopsies before and after decitabine plus camrelizumab combination therapy.

		**Case 1**	**Case 2**	**Case 3**
Gender		M	F	F
Age		47	74	46
Stage at diagnosis		IV, metastatic	IV, metastatic	IV, metastatic
Smoker		Yes	No	No
EGFR mutations		No	L858R, T790M	No
ALK mutations		No	No	No
Before	TMB (Muts/Mb)	7.83	2.49	1.91
	TNB (Neos/Mb)	3.72	1.55	0.75
	MSI (%)	L:1.91	L:2.47	L:4.72
	HLA LOH	HLA-C:03*03	HLA-A:33*03 HLA-B:44*03 HLA-C:07*06	HLA-B:46*01 HLA-C:01*02
	PD-L1 (%)	Positive	NR	Positive
After	TMB (Muts/Mb)	0.1	2.98	1.16
	TNB (Neos/Mb)	0.03	1.32	0.45
	MSI (%)	L:1.50	L:1.91	L:0.78
	HLA LOH	HLA-C:03*03	HLA-A:33*03 HLA-B:44*03 HLA-C:07*06	HLA-B:46*01 HLA-C:01*02
PFS (months)		5.4	6.8	6.5

*PFS, progression free survival; TMB, tumor mutation burden; TNB, tumor neoantigen burden; MSI, microsatellite instability; HLA LOH, HLA loss of heterozygous*.

## Discussion

We report three patients with advanced NSCLC who failed to respond to first-line treatment. The patients had successfully completed a novel treatment regimen of low-dose decitabine plus antibodies; however, gene sequencing of the tumor tissues of these three patients revealed a low TMB, low TNB, low MSI, and HLA LOH, which did not contribute to immunotherapy. Effective treatment of these three patients revealed that epigenetic drugs may sensitize PD-1/PD-L1 blockage, exposing new ideas for advanced NSCLC.

In our previous study, we found that the increased IFNγ^+^ T cell infiltration can be responsible for the clinical responses of low-dose decitabine antitumor therapy in solid tumor patients (including four patients with lung adenocarcinoma) (7). Futhermore, we also discovered that low-dose decitabine plus PD-1 inhibitors may reverse resistance to PD-1 inhibitors in patients with relapsed/refractory classical Hodgkin lymphoma patients in whom second-line treatment or higher failed (4). Wrangle et al. found that advanced NSCLC patients who have previously used decitabine can benefit from PD-1/PD-L1 antibodies (11). This suggested that HMAs may enhance and sensitize response to PD-1/PD-L1 blocking therapy in advanced NSCLC. However, in a phase II clinical study on advanced NSCLC, CC-486 (oral azacitidine) did not increase the median progression-free survival (PFS) associated with pembrolizumab (2.9 vs. 4 months). Moreover, CC-486 increased toxicity, which led to treatment discontinuation (12). Unlike the above clinical trial results, the PFS of the three patients in our study were 5.4, 6.8, and 6.5 months, respectively. Concurrently, of these three patients, one patient complained of mild nausea during treatment, one patient admitted to having moderate weakness and vomit, and the other patient complained of occasional mild appetite decreased. Clinicians assessed the adverse events of these three patients according to the CTCAE (version 4.0). The only severe adverse events is anemia, which is tolerated. Controllable treatment toxicity in the three patients did not affect treatment progress. These adverse events also existed in the clinical study of CC-486 combined with pembrolizumab in advanced NSCLC (12)

The discrepancies between the two studies may be partly explained by different epigenetic drugs. Compared with azacitidine, decitabine is associated with higher response rates in myelodysplastic syndromes (13). In addition, the duration of oral administration of cc-484 (300 mg/day, days 1–14) in this clinical study was much longer than that of decitabine (10 mg/day, days 1–5) in our study. Different doses of HMAs may lead to different levels of tolerance in patients, resulting in differences in the effectiveness and safety of treatment. More importantly, HMAs has two-way dose-dependent anti-tumor properties. High-dose HMAs mainly enters DNA by covalently capturing DNA methyltransferase, which leads to the suspension of the cell cycle and cytotoxic effects. However, low-dose HMAs is not a traditional cytotoxic effect, but changes the epigenetic profile of tumor cells. It can prevent the methylation of the tumor suppressor gene promoter CPG island and restore the expression of suppressor genes that affect tumor growth. Therefore, low-dose HMAs can not only avoid excessive cytotoxicity, but also inhibit tumor growth by regulating the epigenetic state (14–16). Due to the rapid clearance of cytidine deaminase in the gut and liver, the oral bioavailability of HMAs is very low, which is one of the reasons for the poor oral effect of HMAs (17). Therefore, intravenous infusion of low-dose decitabine may be more helpful to sensitize PD-1 antibodies against tumors under low toxicity.

Research on the biomarkers of PD-1/PD-L1 blockade is extensive. Numerous studies have shown that PD-L1 positive patients can benefit from PD-1 or PD-L1 blockade (18, 19). However, recent meta-analyses have confirmed that irrespective of whether PD-L1 is negative or positive, it can benefit from PD-1 or PD-L1 blocking antibodies (20, 21). Of the three cases we studied, two were PD-L1 positive and benefited from HMAs combined with PD-1/PD-L1 antibody therapy. Hence, PD-L1 positivity may be a good biomarker for the combination of decitabine and PD-1 inhibitor, but there is still a need for more studies to confirm this finding.

High TMB, high TNB, and high MSI have been shown to be good biomarkers for immunotherapy (22–24), whereas HLA LOH is thought to as an increased risk of resistance to immunotherapy (25). All three patients had low TMB, low TNB, low MSI, and HLA LOH positivity, and it is speculated that patients may not benefit from PD-1 blockade. However, the real-world situation is that all three patients benefited from decitabine combined with PD-1 antibodies, revealing that low-dose decitabine may increase the sensitivity of PD-1 antibody therapy to avoid tumor escape and prevent resistance to immunotherapy, whereas TMB, TNB, MSI, and HLA LOH may not be good biomarkers of low-dose decitabine combined with PD-1 antibodies.

PD-1 antibodies can increase the anti-tumor effect by preventing T cell exhaustion, but the epigenetic profile of exhausted T cells is stable and limits long-term rejuvenation and the antitumor effects of exhausted T cells after PD-1 blockade (26). Concurrently, low-dose decitabine can alter the epigenetic status of tumors and immune cells. Our previous studies have revealed that low-dose decitabine can increase the anti-tumor capacity of T cells in solid tumors by increasing CD8^+^, CD4^+^, and IFNγ^+^ T cell infiltration and broadens the peripheral T cell receptor repertoire (5–7). In addition, decitabine can increase the expression of interferon signaling pathways, antigen presentation pathways, and cytokine pathway-related genes (11).

Therefore, decitabine can sensitize PD-1 antibodies, which is supported by multiple theoretical mechanisms, but further research on the topic is needed.

## Conclusion

We present a novel, successful, immunotherapy attempt for advanced NSCLC in three patients. Although the number of cases is limited and there are no prospective confirmatory data, our findings reveal that epigenetic regulation of immunotherapy is a reasonable suggestion in NSCLC. Besides, our specific drug dosage and duration are also available for reference. Until now, good predictors for epigenetic drugs combined with immune checkpoint blocking therapy are still lacking, and research on their mechanisms is still insufficient. Thus, we propose conducting more clinical studies on combined epigenetic agents and immunotherapy to improve the resistance of immunotherapy for advanced NSCLC and other solid tumors.

## Data Availability Statement

The raw data supporting the conclusions of this article will be made available by the authors, without undue reservation.

## Ethics Statement

The studies involving human participants were reviewed and approved by The Institutional Ethics Committee of Chinese PLA General Hospital. The patients/participants provided their written informed consent to participate in this study. Written informed consent was obtained from the individual(s) for the publication of any potentially identifiable images or data included in this article.

## Author Contributions

KF and WH: conception and design. YZ: gene sequencing. XY, YZ, YL, QY, LD, ZW, JN, DC, MB, DT, KF, and WH: manuscript writing and manuscript review. DC, MB, DT, and KF: manuscript revision. All authors made substantial contributions to the manuscript.

## Conflict of Interest

YZ was employed by the company YuceBio. The remaining authors declare that the research was conducted in the absence of any commercial or financial relationships that could be construed as a potential conflict of interest.
