# Supplementary material for: Case Report: Low-Dose Decitabine Plus Anti-PD-1 Inhibitor Camrelizumab for Previously Treated Advanced Metastatic Non-Small Cell Lung Cancer
Source: Front Oncol. 2020 Oct 22;10:558572. doi: 10.3389/fonc.2020.558572 (PMC7649792; doi:10.3389/fonc.2020.558572)
Supplement: Supplementary file 1 [file Table_1.DOCX]

**Supplementary Material**

**Inclusion and exclusion criteria**

**Study population：**

Eligible subjects should meet all criteria of inclusion and exclusion，details as follows：

**Inclusion Criteria：**

1.Age from 18 to 75 years with estimated life expectancy＞3 months.

2. Histopathological confirmed advanced unresectable or metastatic NSCLC and at least one measurable disease (≥1cm).

3. Subjects should provide fresh tumor tissue samples or formalin-fixed paraffin embedded tumor archival samples within 3 months and willing to accept tumor re-biopsy in the process of the study.

4. Subjects may have received prior radiotherapy, chemotherapy, or other local ablative therapies, which completed ≥4 weeks prior to registration and patient has recovered to ≤grade 1 toxicity.

5.ECOG(Eastern Cooperative Oncology Group) performance status of 0-2.

6.Adequate organ and marrow function obtained ≤2 weeks of treatment initiation as defined below：

Leukocytes greater than or equal to 3.0 x 10^9/L.

Absolute neutrophil count greater than or equal to 1.0 x 10^9/L.

Platelets greater than or equal to 100 x 10^9/L.

Hemoglobin greater than or equal to 90 g/L.

Total bilirubin less than or equal to 2 x ULN.

Serum albumin should be no less than 25g/L.

AL T or AST less than 2 x ULN.

Measured creatinine clearance ≥60 mL per min.

7. Ability to understand and willingness to sign a written informed consent document.

8. Women of child-bearing potential and men must agree to use adequate contraception (hormonal or barrier method of birth control; abstinence) prior to study entry, and up to 120 days after the last dose of the drug.

**Exclusion Criteria:**

1. Active, known or suspected autoimmune diseases.

2. Known brain metastases or active central nervous system (CNS). If patients with CNS metastases were treated with radiotherapy for at least 3 months prior to enrollment and have no central nervous symptoms and are off corticosteroids, they will be eligible but will need a brain MRI prior to enrollment.

3. Subjects are being treated with either corticosteroids (>10 mg daily prednisone equivalent) or other immunosuppressive medications within 14 days of enrollment.

4. Prior therapy with anti-PD-1, anti-PD-L1, or anti-CTLA-4 antibody (including Ipilimumab or any other antibody specifically targeting T-cell co-stimulation or checkpoint pathways).

5. History of severe hypersensitive reactions to other monoclonal antibodies.

6. History of allergy or intolerance to study drug components.

7. Substance abuse, medical, psychological or social conditions that may interfere with the patient’s participation in the study or evaluation of the study results.

8. History or concurrent condition of interstitial lung disease of any grade or severely impaired pulmonary function.

9. Uncontrolled intercurrent illness, including ongoing or active systemic infection, symptomatic congestive heart failure, unstable angina pectoris, cardiac arrhythmia (excluding insignificant sinus bradycardia and sinus tachycardia) or psychiatric illness/social situations and any other illness that would limit compliance with study requirements and jeopardize the safety of the patient.

10. History of human immunodeficiency virus (HIV) infection or acquired immunodeficiency

syndrome (AIDS).

11. Pregnant or breast-feeding. Women of childbearing potential must have a pregnancy test

performed within 7 days before the enrollment, and a negative result must be documented.

12. Previous or concurrent cancer within 3 years prior to treatment start EXCEPT for curatively

treated cervical cancer in situ, non-melanoma skin cancer, superficial bladder tumors [Ta

(non-invasive tumor), Tis (carcinoma in situ) and T1 (tumor invades lamina propria)].

13. V accination within 30 days of study enrollment.

14. Active bleeding or known hemorrhagic tendency.

15. Subjects with unhealed surgical wounds for more than 30 days.

16. Being participating any other trials or withdraw within 4 weeks.
